# Nodular Worm Infections in Wild Non-human Primates and Humans Living in the Sebitoli Area (Kibale National Park, Uganda): Do High Spatial Proximity Favor Zoonotic Transmission?

**DOI:** 10.1371/journal.pntd.0004133

**Published:** 2015-10-09

**Authors:** Marie Cibot, Jacques Guillot, Sophie Lafosse, Céline Bon, Andrew Seguya, Sabrina Krief

**Affiliations:** 1 UMR 7206, Eco-Anthropologie et Ethnobiologie, Muséum national d’Histoire naturelle, Paris, France; 2 UMR 7179, Mécanismes adaptatifs: Des organismes aux communautés, Muséum national d’Histoire naturelle, Paris, France; 3 Great Apes Conservation Project (GACP), Sebitoli Research Station, Kibale National Park, Fort Portal, Uganda; 4 Department of Parasitology, Dynamyc research group EnvA-UPEC, Ecole nationale vétérinaire d’Alfort, UPE, Maisons-Alfort, France; 5 Uganda Wildlife Authority, Kampala, Uganda; Uniformed Services University, UNITED STATES

## Abstract

**Background:**

Nodular *Oesophagostomum* genus nematodes are a major public health concern in some African regions because they can be lethal to humans. Their relatively high prevalence in people has been described in Uganda recently. While non-human primates also harbor *Oesophagostomum* spp., the epidemiology of this oesophagostomosis and the role of these animals as reservoirs of the infection in Eastern Africa are not yet well documented.

**Methodology/Principal Findings:**

The present study aimed to investigate *Oesophagostomum* infection in terms of parasite species diversity, prevalence and load in three non-human primates (*Pan troglodytes*, *Papio anubis*, *Colobus guereza*) and humans living in close proximity in a forested area of Sebitoli, Kibale National Park (KNP), Uganda. The molecular phylogenetic analyses provided the first evidence that humans living in the Sebitoli area harbored *O*. *stephanostomum*, a common species in free-ranging chimpanzees. Chimpanzees were also infected by *O*. *bifurcum*, a common species described in human populations throughout Africa. The recently described *Oesophagostomum* sp. found in colobine monkeys and humans and which was absent from baboons in the neighboring site of Kanyawara in KNP (10 km from Sebitoli), was only found in baboons. Microscopic analyses revealed that the infection prevalence and parasite load in chimpanzees were significantly lower in Kanyawara than in Sebitoli, an area more impacted by human activities at its borders.

**Conclusions/Significance:**

Three different *Oesophagostomum* species circulate in humans and non-human primates in the Sebitoli area and our results confirm the presence of a new genotype of *Oesophagostomum* recently described in Uganda. The high spatiotemporal overlap between humans and chimpanzees in the studied area coupled with the high infection prevalence among chimpanzees represent factors that could increase the risk of transmission for *O*. *stephanostomum* between the two primate species. Finally, the importance of local-scale research for zoonosis risk management is important because environmental disturbance and species contact can differ, leading to different parasitological profiles between sites that are close together within the same forest patches.

## Author Summary

Nodular worms frequently infect primates, pigs and ruminants. These intestinal nematodes induce inflammatory masses in the colon wall that cause severe abdominal pain, diarrhea, weight loss, and potential death. Through microscopic and molecular analyses, we studied the presence of nodular worms in three non-human primates (chimpanzees, baboons, black and white colobus) and humans inhabiting the Sebitoli area, at the extreme north of Kibale National Park in Uganda. Three different *Oesophagostomum* species were identified in the primates studied and we confirmed the existence of a recently described clade in baboons. Because the Sebitoli chimpanzees displayed a high prevalence of infection and because a high spatiotemporal overlap between humans and apes occurred in our study area, the risk of transmission of *O*. *stephanostomum* between the two species cannot be neglected. Thus, our results add to our understanding of nodular worm infection in location where non human primates and humans are co-existing, and underline the necessity to conduct further research at a local scale in a public health concern.

## Introduction

Emerging zoonotic diseases are a serious threat to public health and animal conservation. This is especially true for apes, whose close phylogenetic relationship with humans increases the risk of zoonotic transmission between them. Although humans have always shared habitats with non-human primates, the dynamics of their relationships are rapidly changing nowadays. Indeed, non-human primate populations suffer from forest loss and fragmentation [1–3] and an increasing number of them live in anthropogenically disturbed habitats such as farmlands, human settlements, fragments of forest, and isolated protected areas [4–6]. As a consequence, people and non-human primates live in increasing spatial proximity to each other [7]. So far, several cases of pathogen transmission have been reported to have occurred between non-human primates and humans; these include the transmission of viruses (e.g. [8–10]), bacteria (e.g. [11–13]) as well as blood-borne parasites (e.g. [14–16]), and intestinal parasites (e.g. [17–21]).

Nematodes of the genus *Oesophagostomum* are intestinal parasites, which frequently infect primates (including monkeys, apes and humans), domestic and wild pigs and ruminants [22, 23]. Uninodular oesophagostomosis (i.e. Dapaong tumor) and multinodular oesophagostomosis [24], which are caused by *O*. *bifurcum* in humans [25], have been reported in endemic foci in West Africa (Togo and Ghana) with an estimated 250,000 infected people and a further one million at risk of contracting these parasitic diseases [26]. Third stage larval development in the colon wall induces the aforementioned inflammatory masses that cause severe abdominal pain, diarrhea and weight loss, and occasional death from peritonitis and intestinal occlusion [24]. While a variety of drugs kill *Oesophagostomum* nematodes, these drugs appear to be less effective on the tissue-dwelling stage because of the difficulty they have passing through the nodule wall [25]).

Only nine cases published in six studies before the 1980s [23, 27] and six cases published in one study in 2014 [18] reported the presence of human oesophagostomosis in Uganda. The few reported cases might be related to a low rate of infection. Nevertheless, oesophagostomosis infections may also be underdiagnosed, notably because obtaining a definitive diagnosis by ultrasound examination [26] is rarely undertaken in Ugandan hospitals and dispensaries [18]. Because transmission occurs through ingestion of the infective third-stage larvae [25] present in water, in food or on the ground, oesophagostomosis is a potential zoonotic risk when humans and non-human primates share the same habitats. In Ghana, identification of genetic differences among *Oesophagostomum* nematodes found in different primate hosts suggested that infection with these nematodes was rarely zoonotic [28, 29]. Still, the risk of zoonotic infection from the presence of infected chimpanzees in the vicinity of humans was mentioned in a study conducted in Kibale forest in western Uganda [17], while a recent study undertaken in the same area described a novel *Oesophagostomum* clade that infects humans and five sympatric species of non-human primates [18]. Because of the severity of the clinical consequences of oesophagostomosis, it should not remain a neglected area of public health. Surprisingly, while captive chimpanzees suffer from oesophagostomosis, no evident clinical signs have been observed in wild chimpanzees so far [30], except for one individual from Gombe (Tanzania) who developed weight loss as well as diarrhea prior to death, without other predisposing factors [31]. It has been suggested that ingestion of rough leaves via swallowing might decrease chimpanzee infestation with the parasite. Hairy leaves have a mechanical effect in preventing third stage larvae from penetrating the colon wall and by abrading the intestinal wall thereby leading to the expulsion of the immature and adult worms present in the nodules and in the gut lumen [32]. Wild chimpanzees that harbor these parasites usually do not suffer lethal infections, whereas humans can die from oesophagostomosis.

More specifically, the Sebitoli area, located in the extreme north of Kibale National Park (KNP) in Uganda, is an area with high anthropogenic pressure. Indeed, the human demographic density is high at the forest borders (villages with croplands, tea and eucalyptus plantations, and tea factories) and a tarmac road crosses the forest and the Sebitoli chimpanzee home range [33]. Additionally, the Sebitoli forest was commercially logged in the 1970s [34, 35] and is today mostly degraded and regenerating with 70% of the land cover affected [36]. Studies conducted in Sebitoli but also in KNP and in close-by forest fragments have shown that non-human primates (i.e., chimpanzees, *Pan troglodytes*; baboons, *Papio anubis*; black and white colobus, *Colobus guereza*; redtail monkeys, *Cercopithecus ascanius*; and vervet monkeys–*Chlorocebus pygerythrus*) frequently feed on croplands at the forest edge [37, 38]. At these sites, farmers rank baboons as the worst pests among the non-human primates because they regularly forage in large groups on several different crops at varying stages of maturity [37, 39]. Since then, baboons have come to represent a particularly important risk for people living close to the forest area, equally to chimpanzees, which are less frequent crop raiders but our closest relatives. Also, in the Kibale region, almost 9% of the population has reported direct contact with non-human primates [40] via touching carcasses (60.8% of cases) or butchering these animals (16% of cases); this is important because these activities represent a high-risk for zoonotic transmission of pathogens. In addition to poachers, other humans (e.g. researchers, field assistants and rangers) also regularly enter the forest and are in close proximity with the non-human primates living there. These observations underline the necessity to determine which species pose a risk of transmission in an environment where the risk of zoonosis appears to be particularly important.

This study aimed to investigate *Oesophagostomum* infection in terms of parasite species diversity, rate of infection and parasite loads, in four primate species (humans, chimpanzees–an ape species and closest relative to humans, baboons–a terrestrial monkey species, black and white colobus–an arboreal monkey species) to better understand the zoonotic risk associated with increased spatial proximity locally between humans and wildlife in an area subject to a high rate of environmental disturbance.

## Methods

### Study site

**Fig 1:**
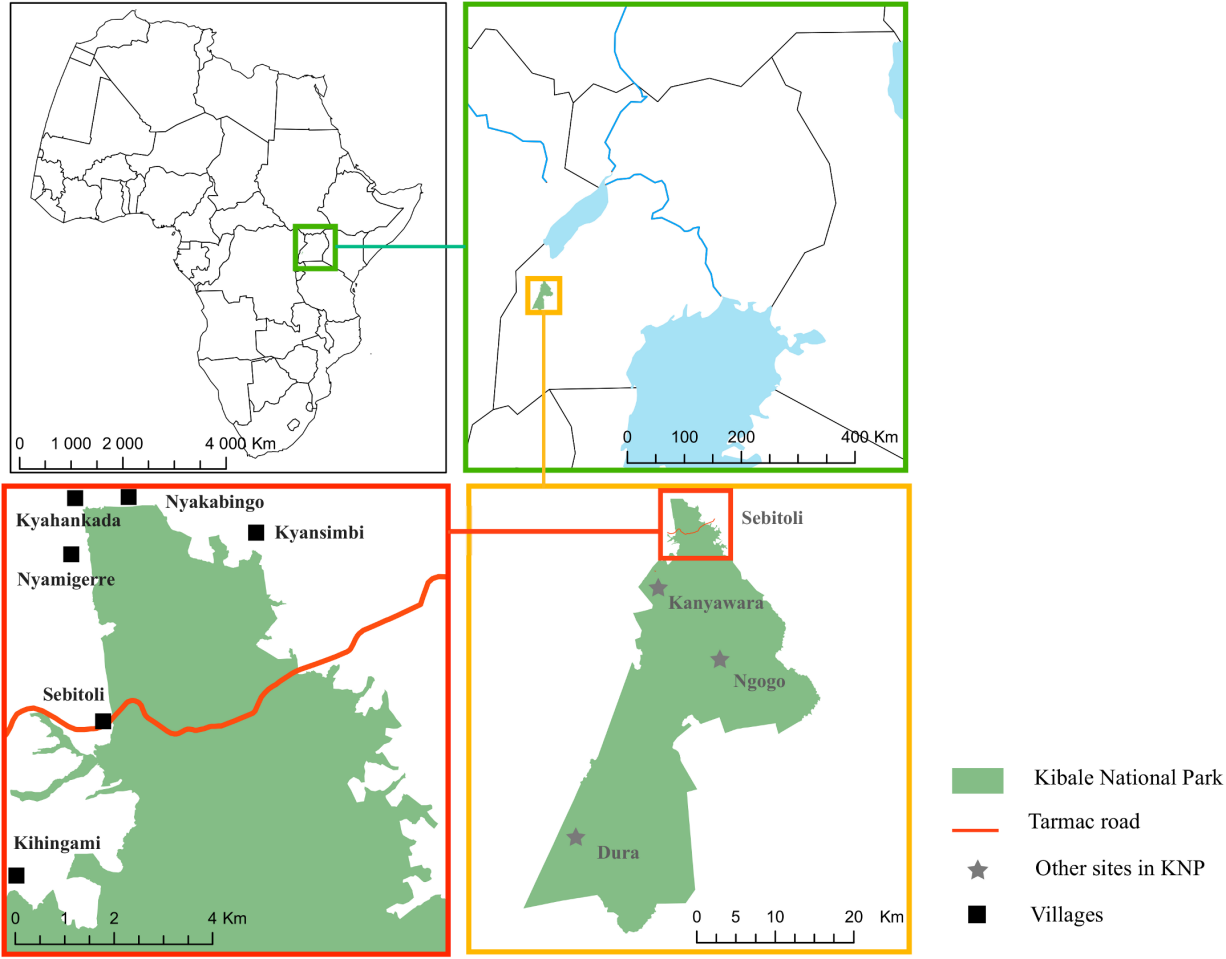
Location of the Sebitoli research area. The area includes the villages sampled and other research sites where infections with *Oesophagostomum* genus have been studied in non-human primates in Kibale National Park, southwestern Uganda.

KNP is located in southwestern Uganda (0°13′ to 0°41′N and 0°19′ to 30°32′E), covers 795 km^2^ [41] and declines in elevation from 1590 m in the north to 1110 m in the south [42], while temperatures in the park range from 23.3 to 24.2°C (annual mean daily maximum; [43]). The park, home of 13 non-human primates species [44], is a mosaic of mature forest (58%), colonizing forest formally used for agriculture (19%), grassland (15%), woodland (6%) and lakes and wetlands (2%) [45] (Fig 1).

At Sebitoli, a long-term research project the “Sebitoli Chimpanzee Project” was initiated in 2008. The chimpanzee habituation level in this area did not allow researchers to collect identified feces from these animals at the time of the study (see below) and other non-human primates are not under habituation. Human pressure around Sebitoli is high. In fact, the human population density within 5 km of the boundary is ~260 inhabitants/km^2^ to the west and 335 inhabitants/km^2^ to the east of the park [46], and 82% of the Sebitoli chimpanzee home range borders are in contact with anthropogenic features [36]. Additionally, an asphalted road with high traffic intensity linking Kampala to the Democratic Republic of Congo crosses the forest [33].

### Sample collections and time periods

Human fecal samples (N = 326) were collected during five different periods (July–August 2010, July–August 2011, March–April 2012, February–March–April 2013, December 2013–January 2014) from people in six villages less than 500 m from the border of the park (Fig 1). Some people were sampled several times at a minimum of 1-month intervals. Participants received instructions on how to collect and store the fecal samples and researchers retrieved them within 1 day of collection.

Fecal samples from baboons (*Papio anubis*) (N = 97) and black and white colobus (*Colobus guereza*) (N = 96) were collected during the first three study periods and fecal samples from chimpanzees (*Pan troglodytes schweinfurthii)* (N = 228) were collected during the five study periods. These samples (< 6 hours old) were collected non-invasively in the forest and immediately transferred to plastic bags. It is likely that samples from the same animals were collected several times because we did not know the identity of the individual from whom the stool was collected. Also, the sample sizes were larger than the number of animals within the groups or community studied. Fecal samples were collected from a relatively large area of the forest (25 km^2^).

Fecal samples from humans and non-human primates were inspected before processing them to check for the presence of macroscopic parasites and to note the consistency (liquid, soft or pasty, solid or normal, and dry or hard, according to a method published previously [47]).

### Coproscopy

Two grams of a fresh fecal sample from a human or a non-human primate was preserved in 18 mL of 10% formalin saline solution. These samples were analyzed at the Department of Parasitology (Ecole Nationale Vétérinaire d’Alfort, France) and a direct microscopic examination of two 50-μL smears was performed to access the presence of hookworm-like eggs (at 100–400x magnification after homogenization). Because collecting fecal samples from different animal species and from humans living in separated villages was time consuming, as well as the fecal sample storage for both microscopy and molecular analyses, we were unable to conduct systematically other microscopic examination methods such as fecal flotation or sedimentation on fresh samples [48]. Each egg was identified according to its size, color, shape and morula aspect. Eggs were not classified at the genus level because the *Oesophagostomum* genus cannot be distinguished with certainty from hookworm nematodes (i.e. *Ancylostoma* sp., *Necator* sp.) by microscopy alone. To establish an arithmetic parasite load (eggs per gram of feces; epg), the total number of eggs, larvae and adults from a 100-μL aliquot was counted and multiplied by 100. Then, a corrected parasite load (CPL) was obtained according to the stool consistency (i.e. x2 when the feces were soft, x3 when the feces were liquid [49]).

### Sample storage for molecular analyses

Fecal samples (N = 15 baboon samples; N = 22 black and white colobus samples; N = 39 chimpanzee samples; N = 39 human samples) were randomly selected and then stored differently according to the study periods: (1) at least 4 g was diluted in 18 mL of 95% ethanol (2010); (2) at least 4 g was diluted in 18 mL of 95% ethanol over a 24 h period, after which the supernatant was removed and the sedimented feces dried on a silica gel beads (2011, 2012); and (3) 10 mL of coproculture products were stored in 50 mL of 95% ethanol (2012, 2013). One gram of fresh feces was mixed with charcoal and vermiculite, and cultured for 10 to 17 days in a Petri dish at room temperature (approximately between 18°C and 26°C). During the culture, regular inspection was done to keep the culture moist and to softly stir the mixture to minimize fungal growth. At the end of the culture, all the mixture was transferred on two layers of gauze and then larvae products were collected via the Baermann procedure, described in [48].

### DNA extraction, PCR and sequencing

Molecular analyses were performed at the Eco-anthropology and Ethnobiology Laboratory (National Museum of Natural History, France). DNA was extracted from coproculture products, feces in ethanol or dried feces. DNA from 10 mL of a larval culture was extracted with a QIAamp DNA Mini Kit Tissue (Qiagen, Chatsworth, CA, USA) according to manufacturer’s protocols but with the following modifications. In step 1, 1 mL of phosphate-buffered saline (PBS) was added to the sample then mixed thoroughly by vortexing, centrifuged and the supernatant removed (3 times), followed by an overnight incubation at -20°C with the ASL buffer included in the kit. DNA was extracted from 100 mg of dried sample or from 2 mL of ethanol sample with a QIAamp DNA Stool Kit (Qiagen). We made a modification to step 1 where 1 mL of PBS was added to the sample, the sample allowed to sit for 10 min at room temperature, and then incubated overnight at 70°C with ASL buffer. An external PCR targeting the ribosomal internal transcribed spacer 2 gene (ITS2) using NC1 and NC2 primers [50], followed by an internal semi-nested PCR using OesophITS2-21 [18] and NC2 primers were performed. PCR reactions were cycled in a BioRad CFX (Bio-Rad Laboratories, Hercules, CA, USA) with a mix of sterile water, Taq polymerase, buffer, primers, dNTPs, fluorochrome and an intercalating agent (Ssofast Evagreen).

The following temperature profile was used for the external PCR: 94°C for 2 min; 45 cycles of 94°C for 10 sec, 60°C for 30 sec, 72°C for 1 min, and a final extension at 72°C for 10 min. The semi-nested PCR followed a slightly different temperature profile: 95°C for 2 min; 45 cycles of 95°C for 10 sec, 55°C for 30 sec, 72°C for 1 min; and a final extension at 72°C for 5 min. The PCR products were sequenced at the Pasteur Institute (Lille, France) by the Genoscreen Laboratory using primer NC2 and OesophITS2-21. DNA sequences were hand-edited and cleaned with 4Peaks software.

### Sequences analysis

Sequence alignments were performed on SeaView software by inputting our sequences with the sequences obtained by Ghai *et al*. [18] (accession numbers: KF250585 –KF250660) and by Krief *et al*. [17] (KT592234, KT592235). In addition, we included three *Oesophagostomum stephanostomum* reference sequences (AF136576, AB821022, AB821031), one *O*. *bifurcum* sequence (AF136575) and five outgroups (HQ844232, Y11736, Y11735, Y10790, AJ006149). Phylogenetic trees were established using the maximum likelihood method in MEGA [51] and the Hasegawa-Kishino-Yano substitution model with five discrete gamma categories [52]. To assess the phylogenetic robustness of the tree, 1000 bootstrap replicates were performed.

### Accession numbers

All the relevant sequences have been deposited in GenBank under the accession numbers: KR149646 –KR149658.

### Sample quality, data analysis and statistics

Special attention was paid to avoiding contamination during all the process stages, that is, during the field collections (utilization of gloves and tongue depressors), during sample storage (use of sterile instruments on different days of collection for each primate species), and during DNA extraction and amplification (separation of the samples by species on the plates and repetitions). The percentage values of the fecal samples that were positive for hookworm-like eggs were considered a proxy for the infection prevalence and the mean corrected parasite load (including infected and non-infected samples) as a proxy for the infection intensity. All statistical tests were performed via R software [53] and were two-tailed with the criterion of statistical significance set at P < 0.05. When samples sizes were small or data were not normally distributed, nonparametric procedures were used.

### Ethics statement

The Uganda National Council for Science and Technology, the Uganda Wildlife Authority and the National Museum of Natural History in France (Memorandum of Understanding SJ 445–12) reviewed and approved the animal care and human research protocols. The free-ranging chimpanzees and monkeys were studied without invasive methods and without interacting with the researchers. Additionally, we obtained the approval of each village chairperson to conduct our research, and human volunteers gave their written informed consent. All volunteers were free to withdraw from the study at any time. The purpose, methods and preliminary findings of the research were explained to all volunteers. Each fresh human sample collected herein was analyzed microscopically within 12h of collection via a fecal flotation to ascertain the parasite species present [49] and to immediately inform the volunteer whether or not he or she had an infection. Following the recommendations of the local dispensary in the area, a single dose anthelmintic treatment (albendazole) was given to any person infected with nematodes (hookworm-like species, *Ascaris lumbricoides* or *Trichuris trichiura*). Persons who received anthelmintic drug treatment could be resampled but at 8-monthly intervals as a minimum.

## Results

### Microscopic examination

**Fig 2:**
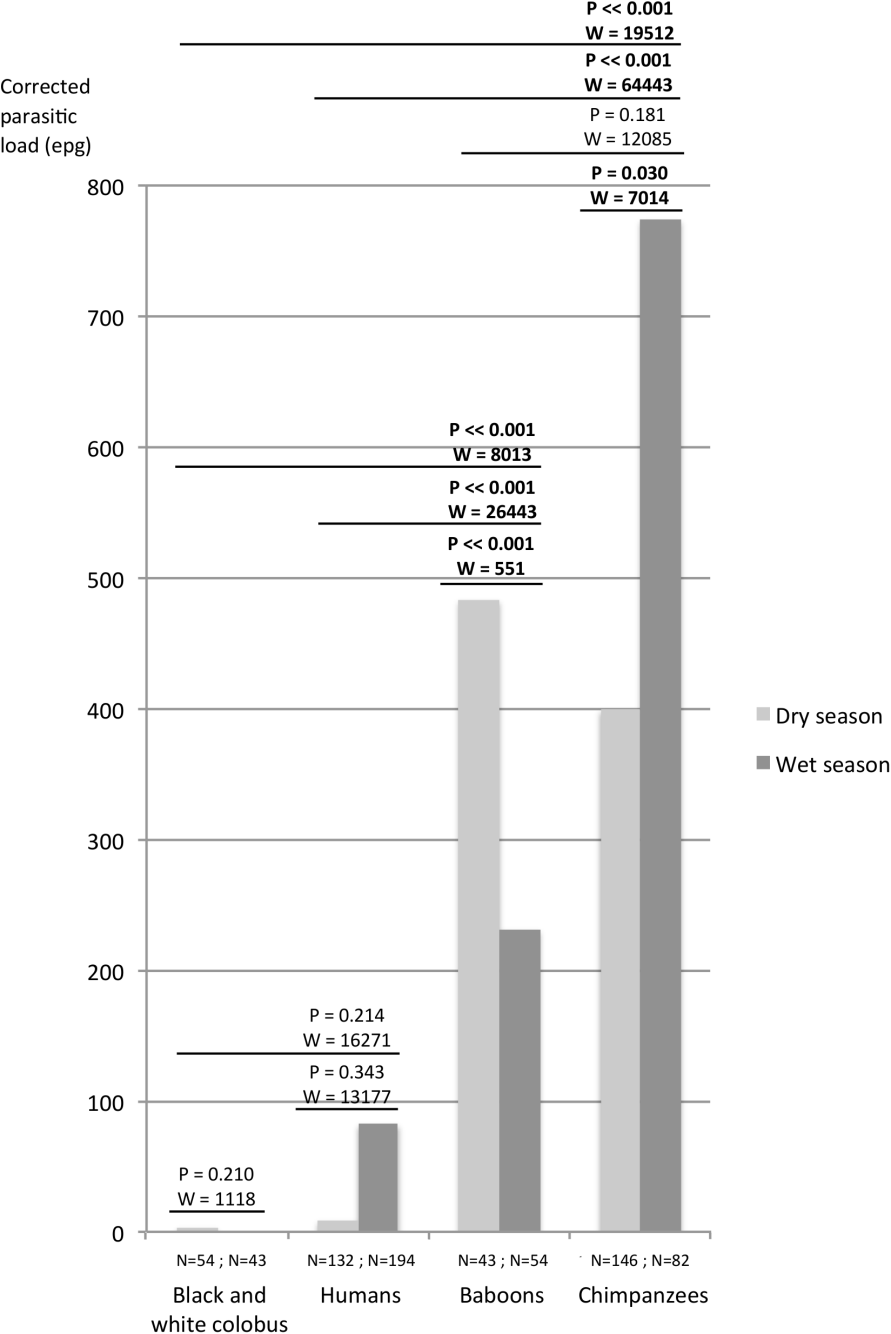
Mean corrected parasitic loads (eggs per gram) according to primate species and according to the season per species (Mann-Whitney tests; NS non significant; * p<0.05; ** p<0.01; *** p<0.001).

The proportions of samples containing hookworm-like eggs varied significantly among the host species (Chi-square = 395.2; df = 3; P<<0.001). Chimpanzees had the highest percentage of positive samples (77.2%; 176/228), followed by baboons (71.1%; 69/97). Humans (6.4%; 21/326) and black and white colobus (2.1%; 2/96) were the two species with low proportions of samples positive in hookworm-like eggs. The mean hookworm-like egg load was also significantly higher in chimpanzee feces (CPL_moy_ = 535 epg) and in baboon feces (CPL_moy_ = 343 epg) compared with human feces (Mann-Whitney tests: W = 64443; P<<0.001 and W = 26443; P<<0.001, respectively) and in black and white colobus feces, whose mean corrected parasite loads were < 100 epg (Mann-Whitney tests: W = 19512; P<<0.001 and W = 8013; P<<0.001, respectively) (Fig 2). During the dry season, the parasite load was higher in baboons than in chimpanzees (Mann-Whitney test: W = 2269; P<0.01) but in the wet season, it was three times higher (statistically significant) in chimpanzees than in baboons (Mann-Whitney test: W = 3071; P<0.001). In comparison with the dry season, during the wet season, the mean hookworm-like egg load was significantly lower in baboon feces (Mann-Whitney test: W = 551; P<<0.001) while it was higher in chimpanzee feces (Mann-Whitney test: W = 7014; P<<0.001) (Fig 2).

### Molecular biology

PCR products from 61 out of the 115 fecal samples tested (92.3% (36/39) of the chimpanzee samples, 93.3% (14/15) of the baboon samples, 36.4% (8/22) of the black and white colobus samples and 28.2% (11/39) of the human samples) produced interpretable DNA sequences. Approximately 50% of the samples stored in ethanol or in ethanol followed by silica gel and more than 75% of the samples that generated coproculture products gave interpretable DNA sequences (Table 1).

**Table 1:** Percentages of samples positive for different parasite species according to the sample storage method.

	Number of samples	O. stephanostomum	O. bifurcum	Oesphagostomum. sp.	Necator sp.	Other parasite species	Total
Ethanol	49	26.9% (14)	16.3% (8)	1.9% (1)	5.8% (3)	1.9% (1)	57.7%
Ethanol-silica gel	26	30.8% (8)	11.5% (3)	3.8% (1)	3.8% (1)	0% (0)	50%
Coproculture	40	48.6% (18)	10% (4)	0% (0)	18.9% (7)	5.4% (2)	75.7%

**Fig 3:**
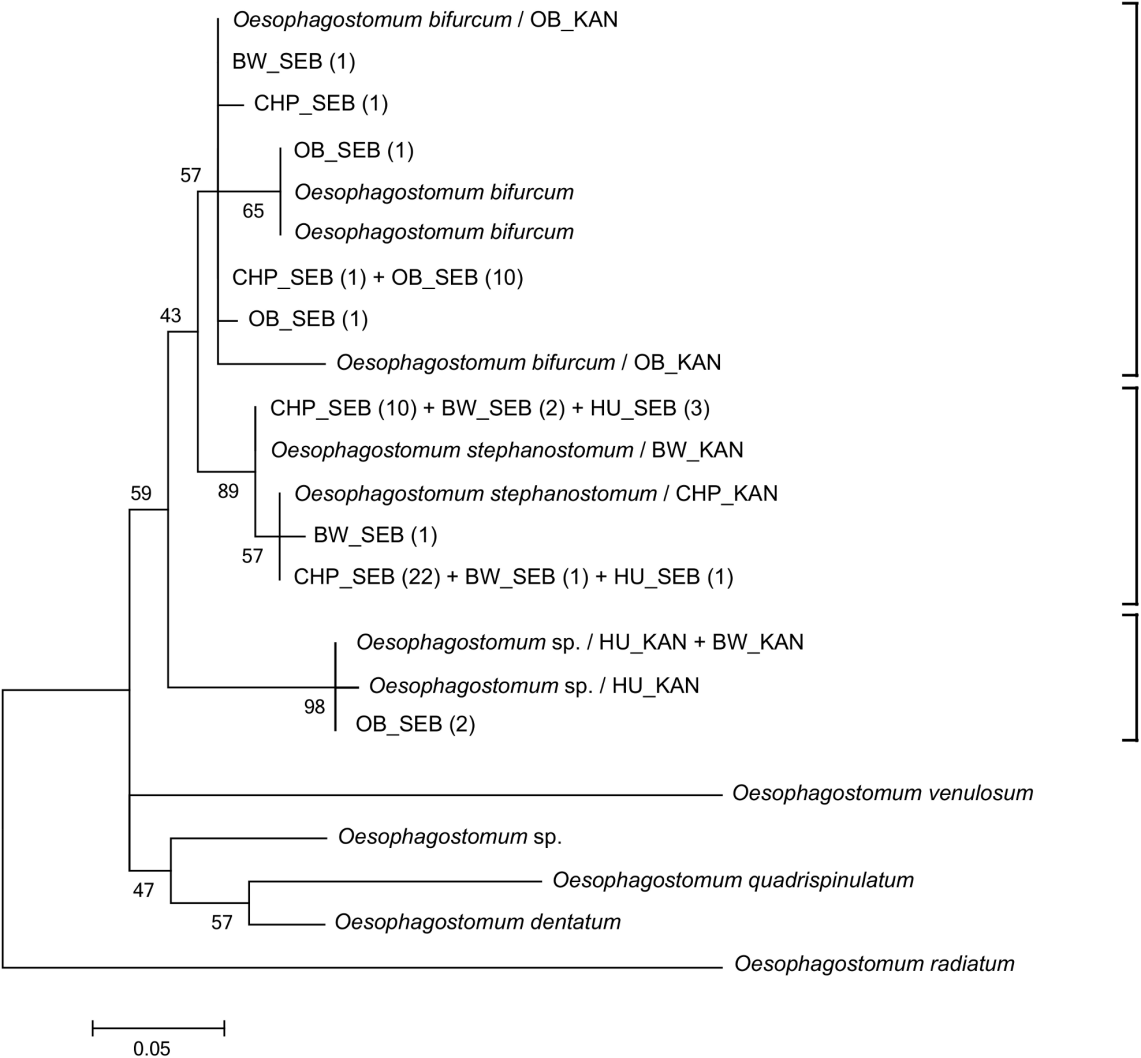
Phylogenetic analysis of *Oesophagostomum* sp. based on ITS2 rDNA (245 bp) sequences. SEB: Sebitoli samples; KAN: Kanyawara samples (according to [17, 18]); HU: humans; OB: olive baboons; BW: black and white colobus; CHP: chimpanzees. The number of infected fecal samples per host species is shown in parentheses. The scale bar indicates the nucleotide substitutions per site. Bootstrap values are shown as percentages.

Fifty-seven DNA sequences (34 from chimpanzees, 14 from baboons, 5 from black and white colobus and 4 from humans) matched the *Oesophagostomum* ITS2 sequences already published. Sequences corresponding to *O*. *stephanostomum* were found in 82.1% (32/39) of the chimpanzee samples, 18.2% (4/22) of the black and white colobus samples and 10.3% (4/39) of the human feces samples. Sequences from one man (60 years of age) and three women (16, 32 and 41 years of age) clustered with the published *O*. *stephanostomum* sequence. Sequences matching with *O*. *bifurcum* were obtained from 80% (12/15) of the baboon samples, from 5.1% (2/39) of the chimpanzee samples and from 4.5% (1/22) of the black and white colobus sample (Fig 3). Only 13.3% (2/15) of the baboon fecal samples were positive for the new sequence type of *Oesophagostomum* sp., recently described in Ghai *et al*. [18] (Fig 3).

### Site comparisons within KNP

Microscopic examinations showed that despite the high infection prevalence in olive baboons and the low infection prevalence in humans being common between sites, Sebitoli black and white colobus monkeys had a low prevalence of infection, as has been described previously in Kanyawara by Gillespie *et al*. [54] but not by Ghai *et al*. [18]. The infection prevalence in chimpanzees and the arithmetic mean corrected for parasite load were significantly higher in Sebitoli than in Kanyawara (Mann-Whitney test: W = 6020, P<0.001) (Table 2).

**Table 2:** Comparisons of the prevalence of hookworm-like eggs and of the corrected parasite loads between different sites within Kibale National Park, Uganda. (1) [55]; (2) [18], (3) [54], (4) [56], (5) [57].

	SEBITOLI	KANYAWARA			NGOGO	DURA
		(1)*	(2)	(3)	(4)	(5)
Method	10% formalin smears	10% formalin smears	Sedimented feces smears	10% formalin smears	10% formalin smears	Fecal flotation
CHIMPANZEES	77.2% (N = 228)	45.8% (N = 203)	-	-	49% (N = 121)	-
	692.6 ± 972.2 epg (N = 176)	431.7 ± 574.3 epg (N = 93)				
BABOONS	71.1% (N = 97)	-	66.7% (N = 27)	-	-	85% (N = 41)
BLACK AND WHITE COLOBUS	2.1% (N = 96)	-	21.6% (N = 37)	6.1% (N = 476)	-	-
HUMANS	6.4% (N = 326)	-	8.3% (N = 36)	-	-	-

* Comparison in parasitic loads between Sebitoli and Kanyawara sites is available for chimpanzees since a same methodology was performed during comparable seasons (*N*_wet/Sebitoli:_ *82 samples; N*_dry/sebitoli_: *146; N*_wet/Kanyawara:_ *90 samples; N*_dry/Kanyawara_: *113*).

At the molecular level, the *Oesophagostomum* clades obtained from chimpanzees in Sebitoli and Kanyawara were similar in terms of the predominance of *O*. *stephanostomum* and *O*. *bifurcum* (less frequent) (Table 3). All of the Kanyawara baboon samples were positive for the *O*. *bifurcum* clade. In addition to *O*. *bifurcum*, the newly described *Oesophagostomum* clade was identified in 14.3% of the Sebitoli baboon fecal samples (Table 3). The rate of *Oesophagostomum* infestation of human feces and colobus feces was twice as low in Sebitoli as in Kanyawara. Specifically, 60% of the *Oesophagostomum* species in Kanyawara black and white colobus monkeys comprised the newly described *Oesophagostomum* sp. clade [18], whereas 80% were *O*. *stephanostomum* in Sebitoli colobine monkeys but *Oesophagostomum* sp. was not detected. Humans living in the Sebitoli area harbored only *O*. *stephanostomum*, while villagers from the Kanyawara area were only infected by *Oesophagostomum* sp. (Table 3).

**Table 3:** Prevalence in *Oesophagostomum* species and subspecies at Sebitoli and Kanyawara as determined by PCR. (1) [17]; (2) [18].

	SEBITOLI	KANYAWARA
CHIMPANZEES		
Oesophagostomum spp.	87.2% (34/39)	83.3% (15/18) (1)
O. stephanostomum	94.1% (32/34)	73.3% (11/15) *
O. bifurcum	5.9% (2/34)	33.3% (5/15) *
Oesophagostomum sp.	0% (0/34)	0% (0/15)
BABOONS		
Oesophagostomum spp.	93.3% (14/15)	100% (27/27) (2)
O. stephanostomum	0% (0/14)	0% (0/12)
O. bifurcum	85.7% (12/14)	100% (12/12)
Oesophagostomum sp.	14.3% (2/14)	0% (0/12)
BLACK AND WHITE COLOBUS		
Oesophagostomum spp.	22.7% (5/22)	56.8% (21/37) (2)
O. stephanostomum	80.0% (4/5)	40% (2/5)
O. bifurcum	20.0% (1/5)	0% (0/5)
Oesophagostomum sp.	0% (0/5)	60% (3/5)
HUMANS		
Oesophagostomum spp.	10.3% (4/39)	25% (9/36) (2)
O. stephanostomum	100% (4/4)	0% (0/6)
O. bifurcum	0% (0/4)	0% (0/6)
Oesophagostomum sp.	0% (0/4)	100% (6/6)

* One chimpanzee was co-infected by both *O*. *stephanostomum* and *O*. *bifurcum*.

## Discussion

In the present study, microscopic and molecular approaches were used to reveal the prevalence and parasitological load of *Oesophagostomum* sp. in three non-human primate species and humans living in close proximity in a forested area. Our results provide the first evidence that some humans living in the Sebitoli area are infected by *O*. *stephanostomum*, a common species in free-ranging chimpanzees. Moreover, the chimpanzees also harboured *O*. *bifurcum*, a species commonly described in humans. Finally, the existence of the new clade *Oesophagostomum* sp. described in black and white colobus monkeys and humans in Kanyawara (a neighboring site to Sebitoli) by Ghai *et al*. [18], was confirmed in the Sebitoli region as two baboon fecal samples were infected with it. Microscopy revealed that the infection prevalence and the parasite load were significantly higher in the Sebitoli chimpanzees than in the Kanyawara ones.

Sebitoli chimpanzees had a high infection prevalence compared with colobine monkeys and humans. While the *Oesophagostomum* species isolated from Sebitoli and Kanyawara chimpanzees are similar, both the prevalence of infection and the corrected arithmetic mean parasite load were higher in the Sebitoli apes. Without additional data, it appears to be difficult to attribute these differences to specific causes. Indeed, individuals may differ in terms of infection rates and parasitic loads according to demography (1.5 individuals/km^2^ at Kanyawara vs. 3.2 individuals/km^2^ at Sebitoli, leading to increased transmission among individuals; [58, 59]) and behaviour (e.g. higher association strength between members of the same community with increased grooming sessions; [60]). These observations might also result from a difference in the chimpanzees’ physiology, immunity, and environment (e.g. difference in proximity to humans and their livestock). Because of their proximity to humans, Sebitoli chimpanzees are more affected by stress, which can be evidence through increased signs of anxiety when chimpanzees leave the forest to crop raid at the borders, and when they cross a tarmac road with high traffic cutting their home range [33, 38]). Stress could decrease their immunity level [61, 62], and thereby make them more susceptible to parasitic infections. Additionally, about 30% of the chimpanzees have limb deformities caused by poaching, 10% of the individuals suffer from facial dysplasia [63] and one of them has a cleft lip [64]. Such mutilations and congenital diseases—suspected to be caused by prenatal exposure to teratogen chemicals—may be associated with other health disorders in the affected individuals and decrease individual’s immunity to pathogens such as parasites [65–67]. Other individuals—without abnormal phenotypes—may also experience effects of such exposure.

A similar prevalence of *Oesophagostomum* spp. and other hookworms was observed in villagers living in Sebitoli and Kanyawara (28.2% of the Sebitoli villagers and 25% of the Kanyawara villagers), but two different *Oesophagostomum* clades were distinguished: *O*. *stephanostomum* in Sebitoli and *Oesophagostomum* sp. in Kanyawara. *O*. *stephanostomum* is common in non-human primates, particularly great apes [17, 18]. In the present study, humans infected with *O*. *stephanostomum* came from Sebitoli village (one man and two women) and from Kyansimbi village (one woman). They were used to seeing chimpanzees and baboons in their gardens, at locations less than 500 m from the forest edge. Interestingly, one of them was used to sleeping in a small hut to prevent animal crop raiding during the night. However, Sebitoli chimpanzees often feed in maize gardens in large parties and they can stay a long time in croplands [38], likely contaminating the fields when defecating. In addition, maize begins to ripen and be consumed by both humans and chimpanzees at the end of the wet season (Cibot *et al*., *submitted*) when the rate of *Oesophagostomum* infection in chimpanzees was the highest. This period poses the highest risk of pathogen transmission through the fecal-oral route. Taken together, these results and observations showing a high degree of spatiotemporal overlap between humans and chimpanzees, which are phylogenetically close species, represent factors that could enhance the risk of transmission for *O*. *stephanostomum* between chimpanzees and people.

In this study, black and white colobus had a low prevalence of infection, which could be related to their arboreality (making them less vulnerable to nematode parasites with a life cycle including soil [18, 68]) and to their diet (important ingestion of secondary compounds in leaves with potential anthelminthic properties [30]), and they are rarely in contact with humans (Cibot *et al*., *submitted*). While oesophagostomosis lesions in baboons are identical to the ones described in chimpanzees and humans (nodules in the intestinal wall; [31, 69]), *O*. *bifurcum*, which appears to be the most common species in Sebitoli baboons, was not found in any of the villagers. Nevertheless, because Sebitoli baboons are reported to be the most frequent crop raiders (Cibot *et al*., *submitted*) and two baboon fecal samples were infected with the new *Oesophagostomum* sp. clade described in humans from Kanyawara, we still cannot exclude the potential disease transmission between the two species. Surprisingly, in baboons, the mean corrected parasitic load of hookworm-like eggs was higher during the dry season compared than it was in the wet season (the opposite of what was observed in humans and chimpanzees in this study). Indeed, lower ambient temperatures and higher humidity rates likely favor survival of eggs and larvae in feces, increasing the risk of infection during the wet season [70]. A long-term survey with an increased sample set should be initiated to confirm this result and investigate why an opposite seasonality pattern exists between chimpanzees and baboons.

Our findings raised the need for better public health awareness of oesophagostomosis in the Kibale region. Further studies should be conducted to better understand the epidemiology of *Oesophagostomum* infections in Uganda, and the role played by domestic animals (cows, sheep, goats, pigs), and other wild animals (antelopes, buffalos, wild pigs) in its transmission. Indeed, a recent study undertaken in Tanzania revealed that crop fields regularly used by both chimpanzees and domesticated animals represented potential hotspots for *Cryptosporidium* transmission [71]. However, when comparing studies, we should interpret carefully data because different methods may have been used. Indeed, in the present study, we detected *N*. *americanus* using the OesophITS2-21 primer, which was supposed to be specific and only allow amplification of the *Oesophagostomum* genus [18]. This result could be caused in part by a difference in the storage procedure between the two studies, with coproculture storage likely favoring the amplification of *Necator* sp. Similarly, we need to be cautious with the prevalence we obtained after PCR and sequencing since we amplified materials issued from different methods and we sampled unidentified individuals for wild primates compared to other studies. Indeed, the relatively low prevalence in *Oesophagostomum* spp. in humans and in colobine monkeys could result from an underestimation of all the nematode species or of certain species of *Oesophagostomum*, due to the culture of fecal samples, which could allow differential development of larva-stage nematodes and which is less sensitive than other methods (e.g. agar plate culture, which requires sterilization systems that are not available in field settings). Then, we likely underestimate the public health problem. Finally, we should also remain prudent when we employ the term “species” in our study, since DNA amplification was only based on a short region of a single gene. While we demonstrated a relatively high genetic diversity within the *Oesophagostomum* genus, the sequencing of additional genes or the morphological identification of the third stage larvae and adults worms should be established to confirm the species level differentiation.

As Bortolamiol *et al*. [36], comparing three sites within the same park including Sebitoli and Kanyawara, revealed that small-scale analysis was needed to obtain a better understanding of chimpanzee diet, repartition, density and land use, the parasitological profiles were different between sites and showed that research is required at a more local scale for zoonosis management. For example, the higher prevalence of *Oesophagostomum* spp. in colobine monkeys observed in Kanyawara compared to our present study may be explained by a difference in the *Oesophagostomum* species harbored in the black and white colobus monkeys, which could have consequences for zoonosis management. Moreover, working at a small scale is also essential for the human-wildlife zoonotic management as local knowledge and traditional beliefs can differ significantly between people living in relatively close locations and even within the same villages. In fact, near the KNP area, different ethnicities live within the same villages [37] and an increasing number of migrants, notably Congolese people fleeing conflicts in the Democratic Republic of Congo, join western Uganda [72]. Migrants may have a different perception of the risks associated with living in close proximity to wild animals, or the risks associated with hunting animals and eating bush meat [40, 73]. In any case, health-risk education programs should be better integrated into conservation programs and measures against crop-raiding and other practices such as throwing food from passing vehicles on the Sebitoli road to feed baboons should be implemented. It should also be important to better follow Ugandan patients in hospitals and dispensaries with clinical examination and ultrasonography to better evaluate impacts on human health. Today, in Northern Togo and Ghana, the *Oesophagostomum* infections seem to have been eradicated after a large scale and intense mass treatment on humans [23]. Finally, our findings reinforce the fact that zoonotic parasites, in the context of increased proximity between non-human primates and humans, should be considered a priority concern for researchers, wildlife managers and health care systems.
